# Prostaglandin Receptor Signaling in Disease

**DOI:** 10.1100/tsw.2007.182

**Published:** 2007-09-01

**Authors:** Toshiyuki Matsuoka, Shuh Narumiya

**Affiliations:** Department of Pharmacology, Faculty of Medicine, Kyoto University, Kyoto 606-8501, Japan

**Keywords:** prostaglandin, prostanoid, thromboxane, receptor, signaling, disease, inflammation, pain, fever, arthritis, asthma, atopic dermatitis, pulmonary fibrosis, immune response, dendritic cell, lymphocyte

## Abstract

Prostanoids, consisting of the prostaglandins (PGs) and the thromboxanes (TXs), are a group of lipid mediators formed in response to various stimuli. They include PGD_2_, PGE_2_, PGF_2α_, PGI_2_, and TXA_2_. They are released outside of the cells immediately after synthesis, and exert their actions by binding to a G-protein coupled rhodopsin-type receptor on the surface of target cells. There are eight types of the prostanoid receptors conserved in mammals from mouse to human. They are the PGD receptor (DP), four subtypes of the PGE receptor (EP_1_, EP_2_, EP_3_, and EP_4_), the PGF receptor (FP), PGI receptor (IP), and TXA receptor (TP). Recently, mice deficient in each of these prostanoid receptors were generated and subjected to various experimental models of disease. These studies have revealed the roles of PG receptor signaling in various pathological conditions, and suggest that selective manipulation of the prostanoid receptors may be beneficial in treatment of the pathological conditions. Here we review these recent findings of roles of prostanoid receptor signaling and their therapeutic implications.

## INTRODUCTION

When tissues are exposed to diverse physiological and pathological stimuli, arachidonic acid (AA) is liberated from membrane phospholipids and is converted to prostanoids, including the prostaglandins (PGs) and the thromboxanes (TXs), by the action of cyclooxygenase (COX). The COX reaction converts AA to an unstable endoperoxide intermediate, $PGH_2$, which, in turn, is metabolized to $PGD_2$, $PGE_2$, $PGF_{2\alpha}$, $PGI_2$, and $TXA_2$ by cell-specific respective isomerases and synthases. Prostanoids thus formed are immediately released outside of the cell. Because they are either chemically or metabolically unstable, it is believed that prostanoids work only locally, near their site of production. Prostanoids exert their actions via membrane receptors on the surface of target cells. A family of membrane receptors mediating their actions has been characterized and cloned.

There are eight types and subtypes of receptors for prostanoid that are conserved in mammals from mouse to human[1]: the PGD receptor (DP), four subtypes of the PGE receptor ($EP_1$, $EP_2$, $EP_3$, and $EP_4$), the PGF receptor (FP), the PGI receptor (IP), and the TXA receptor (TP). All are G-protein coupled rhodopsin-type receptors with seven transmembrane domains, and each is encoded by different genes. Despite the presence of conserved sequences, the overall homology among prostanoid receptors is not high, ranging from approximately 20 to 30%. However, the homology among receptor homologues from various species is considerably higher. The amino acids and nucleotides sequence homology between human and mouse DP, $EP_1$, $EP_2$, $EP_3$, $EP_4$, FP, IP, and TP is 73, 84, 88, 84, 88, 89, 79, and 76%, respectively. In addition, there are several splice variants of the $EP_3$, FP, and TP receptors, which differ only in their C-terminal tails. Thus far, three isoforms of the mouse $EP_3$ and eight of the human $EP_3$ have been identified, two of the human TP have been cloned, and for the FP two from sheep. Among the eight types and subtypes, the IP, DP, $EP_2$, and $EP_4$ receptors mediate a cAMP rise and have been termed "relaxant" receptors, whereas the TP, FP, and $EP_1$ receptors induce calcium mobilization and constitute a "contractile" receptor group. The remaining receptor, $EP_3$, induces a decline in cAMP levels and has been termed the "inhibitory" receptor. However, the effects of prostanoids on these G-protein coupled signaling pathways may change as a function of ligand concentration or structure.

In addition to this family of prostanoid receptors, there is a distinct type of PGD receptor, chemoattractant receptor-homologous molecule expressed on T helper type 2 (Th2) cells (CRTH2)[2]. This receptor was originally cloned as an orphan receptor expressed in Th2 lymphocytes, and has recently been shown to bind $PGD_2$ with an affinity as high as that of DP, though the binding profile to other PGD analogs differs from that of DP. The CRTH2 receptor belongs to the family of chemokine receptors, and mediates chemotaxis to $PGD_2$ of Th2 lymphocytes as well as eosinophils or basophils.

The roles of PGs and their receptor signaling in various physiological and pathophysiological conditions have been examined by comparing the effects of aspirin-like drugs with those of each prostanoid added exogenously. However, such studies do not clearly indicate which type of prostanoid and which class of prostanoid receptor is involved in a given process, nor how critical the actions of prostanoids might be. To address these questions, mice deficient in each prostanoid receptor have been generated and analyzed. In addition, highly selective agonists and antagonists for the cloned receptors have been developed, although there are species' differences in their actions. In this article, we review the recent findings obtained from the analyses of these animals in various disease models, and discuss the roles of PG receptor signaling in pathological conditions. A special focus is given to immune and inflammatory responses.

## ACUTE INFLAMMATION

Acute inflammation is the early response to tissue injury, infection, or immunological challenge. Local reddening, heat generation, swelling, and pain are classic signs of acute inflammation. These symptoms, except pain, are caused by increased blood flow and vascular permeability. Previous studies suggested that prostanoids contribute to these processes by causing peripheral vasodilation and synergize with other mediators, such as histamine and bradykinin, to cause an increase in vascular permeability and edema[3]. Among the various PG species, $PGI_2$ and $PGE_2$ have been implicated as the PGs most responsible for inflammation because of their abundance in inflammatory exudates and tissues[4,5,6,7] and their ability to mimic inflammatory responses by their administration *in vivo*.

Murata et al.[8] employed carrageenan-induced paw swelling as a model for acute inflammation. In this model, swelling increased in a time-dependent manner up to 6 h after injection and was decreased by about 50% on treatment with indomethacin. $IP^{-/-}$ mice developed swelling only to a level comparable to that observed in indomethacin-treated wild-type (WT) mice, and indomethacin treatment of $IP^{-/-}$ animals did not induce a further decrease in swelling. This study clearly demonstrated that $PGI_2$-IP signaling mediated this type of acute inflammation physiologically. However, it remained to be examined whether $PGI_2$-IP and/or $PGE_2$-EPs signaling have different context-dependent roles in other types of inflammation.

Yuhki et al.[9] addressed this issue with a mouse pleurisy model. Pleurisy has been used to evaluate edema formation with both the rate and degree of exudates formation[10,11]. In this model, the volume of the pleural exudate increased with a peak around 3 to 5 h after the carrageenin injection, and total leukocytes in the exudates also increased to reach a plateau at 5 h and remained constant up to 24 h. The main PGs in the exudates were 6-keto-$PGF_1$, a stable metabolite of $PGI_2$, and $PGE_2$. Given the significantly less exudate volume in $IP^{-/-}$, $EP_2^{-/-}$, and $EP_3^{-/-}$ mice than that in WT mice, participation of $EP_2$ and $EP_3$ along with IP in exudate formation were revealed. They could not examine the roles of prostanoid receptors in leukocyte migration, as leukocyte number was not influenced by indomethacin treatment in this model. This feature was very different from the reported case in rats that leukocyte migration was suppressed by indomethacin[12,13,14]. Given the different regulation of chemokine or cytokine induction by the prostanoids between species and/or strains, the precise mechanism leading to leukocyte migration remains to be determined. They also performed RT-PCR for IP, $EP_1$, $EP_2$, $EP_3$, and $EP_4$ in the tissues around the pleural cavity and detected mRNA expressions of all receptors except $EP_1$. At 3 h of carrageenin injection, the expression levels of all these mRNA decreased in the pleura, while there was no apparent change in diaphragm and lung. The reason for the decreased mRNA and the precise localization of these receptors remain to be clarified. In consideration of the signal transduction, cAMP increase after the activation of either IP or $EP_2$ by coupling to Gs protein are expected to be responsible for the exudate formation; the responsible $EP_3$ receptor isoform and coupled G-protein should be determined in the future investigation, because there are several isoform receptors for $EP_3$, all of which have different signaling.

Goulet et al.[15] have examined another series of mice lacking prostanoid receptors in an experimental model, AA-induced cutaneous inflammation, that depends primarily on the synthesis and release of eicosanoids. In this model, they found that COX-1, but not COX-2, has the capacity to induce acute inflammation on the initiation phase and that this response is mediated by $PGE_2$-$EP_3$ signaling. However, they observed these mice only during the early phase, 1 h after AA application on mice ears. The contribution of prostanoid receptors in a later phase of inflammation remains to be characterized.

Thus, these studies have revealed the critical PG receptor signaling pathways for promoting acute inflammation and the context-dependent effects of this signaling, which could be determined by the types and the phases of inflammation. However, there is also evidence to support a role for prostanoids in the resolution of inflammatory responses[16]. It should be noted that the profiles of prostanoids generated in an inflammatory site change during the course of inflammation, and are also dependent on the stimulus and site of inflammation.

## PAIN

The role of PGs in inflammatory pain is also well accepted because of the antinociceptive effects of aspirin-like drugs. Previous studies reported that PGs added exogenously are able to induce hyperalgesia, an increased sensitivity to a painful stimulus, or allodynia, a pain response to a usually nonpainful stimulus, in various model systems. These studies using exogenous PGs showed that $PGE_2$, $PGE_1$, and $PGI_2$ exert stronger effects than the other types of PGs, indicating the involvement of these two signaling pathways via EP or IP receptors in inducing inflammatory pain[17]. The main site of hyperalgesic action of prostanoids lies in the periphery where PGs are believed to sensitize the free ends of sensory neurons. The primary sensory afferents have their cell bodies in the dorsal root ganglion (DRG), and several types of prostanoid receptor mRNAs, including those of IP, $EP_1$, $EP_3$, and $EP_4$, were found in neurons in the ganglion[18,19]. Thus, these PG signaling pathways have been suggested to mediate pain.

Murata et al.[8] used $IP^{-/-}$ mice to address this issue. $IP^{-/-}$ mice did not show any alteration in their nociceptive reflexes examined by hot plate and tail flick tests, indicating that $PGI_2$ is not involved in nociceptive neurotransmission at the spinal and supraspinal levels. On the other hand, when these mice were subjected to the acetic acid–induced writhing test, they showed markedly decreased responses compared with control WT mice, and their responses were as low as those observed in control mice treated with indomethacin. Additionally, both $PGE_2$ and $PGI_2$ injected intraperitoneally induced modest writhing responses in WT mice, whereas $IP^{-/-}$ mice showed responses only to $PGE_2$. These results indicate that the hyperalgesic response in this model is evoked by $PGI_2$-IP signaling in the peripheral end of nociceptive afferents.

However, $EP_1$ involvement in mediating pain is also suggested by Stock et al.[20], not only in the acetic acid–induced writhing test, but also in the 2-phenyl-1, 4-benzoquinone–induced (PBQ-induced) stretch assays, as reduced responses were observed in their $EP_1^{-/-}$ mice. Their pain sensitivity responses were reduced by approximately 50%, and this reduction in the perception of pain was virtually identical to that in WT mice using piroxicam, a COX inhibitor. While the reason for these conflicting results is elusive, the difference in genetic background between the $IP^{-/-}$ and $EP_1^{-/-}$ mouse lines is one factor that may contribute. Alternatively, both the $PGE_2$-$EP_1$ and $PGI_2$-IP contribute to inflammatory pain, and the absence of either signaling is sufficient to attenuate the response. The involvement of these two signaling in hyperalgesia was also reported by the analyses of their roles in the sensitization of capsaicin receptor TRPV1, a nonselective cation channel expressed in sensory neurons and activated by various noxious stimuli. Both $PGE_2$-$EP_1$ and $PGI_2$-IP signaling potentiate TRPV1 activity in a protein kinase C (PKC)–dependent manner[21].

Recently, it has become clear that inflammatory and immune mechanisms, both in the periphery and the central nervous system, play an important role in pain[22]. Ueno et al.[23] demonstrated that the receptors other than IP work to amplify pain sensations in pathological conditions. They pretreated $EP_1^{-/-}$, $EP_2^{-/-}$, $EP_3^{-/-}$, $EP_4^{-/-}$, $IP^{-/-}$, and WT mice with lipopolysaccharide (LPS), and then examined their hyperalgesic responses in acetic acid administration. Whereas $EP_1^{-/-}$, $EP_2^{-/-}$, and $EP_4^{-/-}$ mice showed a similar enhanced writhing response as the WT mice, $IP^{-/-}$ and $EP_3^{-/-}$ mice showed significant reductions. Thus, the nociception of the writhing response in LPS-pretreated mice is mediated by both IP and $EP_3$ receptors. They conclude that the nociception of the writhing response in non-LPS–treated mice could be mediated mainly by $PGI_2$-IP signaling, and that the perception of enhanced pain in LPS-pretreated mice requires the mediation of both $PGI_2$-IP and $PGE_2$-$EP_3$ signaling.

$PGE_2$-$EP_2$ signaling is also reported to contribute to the spinal inflammatory hyperalgesia in the zymosan A peripheral inflammation model[24]. In this model, the yeast extract zymosan A was injected subcutaneously into the plantar side of the left hind paw to induce inflammatory hyperalgesia. In WT mice, paw withdrawal latencies decreased within 4 h, remained stable for about 24 h, and then recovered slowly within 7 days. In $EP_2^{-/-}$ mice, thermal and mechanical sensitization was very similar to that seen in WT mice at 2 h. However, from 4 h onward, $EP_2^{-/-}$ mice recovered much faster from hyperalgesia, and a significant difference between WT mice and $EP2^{-/-}$ mice became obvious at 4–6 h. From day 3–4 onward, thermal hyperalgesia in $EP_2^{-/-}$ mice became statistically indistinguishable from that in vehicle-injected control mice. Similar effects were obtained for mechanical hyperalgesia. Thus, $EP_2^{-/-}$ mice exhibited an almost normal early hyperalgesia, but completely recovered from sensitization within 2 days, indicating a dominant role of $PGE_2$-$EP_2$ signaling in the generation of inflammatory pain.

Involvement of $PGE_2$-$EP_4$ signaling in pain sensitization is also demonstrated in rat[25]. It was shown that $EP_4$ receptor subtype is expressed by a subset of primary sensory DRG neurons, and that its levels, but not that of the other $EP_{1–3}$ subtypes, increase in the DRG after complete Freund's adjuvant–induced peripheral inflammation. Administration of both an $EP_4$ antagonist and $EP_4$ knockdown attenuates inflammation-induced thermal and mechanical hypersensitivity, without changing basal pain sensitivity.

In addition to these hyperalgesic actions, PGs are also involved in allodynia, augmenting processing of pain information in the spinal cord. Minami et al.[26] found that intrathecal injection of $PGE_2$ into mice induced allodynia; the mice showed squeaking, biting, and scratching in response to low-threshold stimuli. To characterize EP subtypes involved in $PGE_2$-induced allodynia, they examined the response in the $EP_1^{-/-}$ or $EP_3^{-/-}$ mice[27]. Intrathecal (i.t.) administration of $PGE_2$ induced allodynia over the 50-min experimental period in the WT and the $EP_3^{-/-}$ mice, but not in the $EP_1^{-/-}$ mice, suggesting that the $EP_1$ receptor is involved in the $PGE_2$-induced allodynia. Eguchi et al.[28] examined further $PGE_2$-induced allodynia in mice deficient in lipocalin-type PGD synthase. They found that $PGE_2$-induecd allodynia was absent in these mice, and was recovered by the i.t. addition of $PGD_2$. Interestingly, the recovering effect of $PGD_2$ showed a bell-shaped dose-effect relationship, suggesting that $PGD_2$ exerts dual actions in elicitation of allodynia by $PGE_2$. The PGD synthase-deficient mice also exhibited loss of allodynia induced by i.t. injection of a γ-aminobutyric acid (GABA) antagonist, bicuculline, indicating that $PGD_2$ also mediated this type of allodynia.

Recent studies of prostanoid receptor mutant mice have provided a lot of clear evidence that PG receptor signaling mediates pain. However, more detailed analyses of the complex mechanisms of pain regulation at the molecular levels are required for the comprehensive understanding of their involvement, since the different main signaling pathways have been shown to mediate pain in a context-dependent manner.

## FEVER AND OTHER MANIFESTATIONS OF SYSTEMIC ILLNESS

Systemic illness is associated with fever, adrenocorticotropic hormone (ACTH) release, loss of appetite and libido, decreased locomotion, and the induction of slow-wave sleep. Because of the antipyretic action of aspirin-like drugs, it has long been speculated that prostanoids, especially $PGE_2$, are a principal mediator of fever[29]. Systemic administration of LPS, a bacterial endotoxin, is thought to produce fever by inducing COX-2, a rate limiting enzyme for $PGE_2$ synthesis, by both venular endothelial cells and perivascular microglial cells[30,31,32,33]. $PGE_2$ released into the brain may act on the neurons expressing EP receptors in the anteromedial preoptic area of the hypothalamus (POA)[34], where expression of $EP_1$, $EP_3$, and $EP_4$ receptors are demonstrated in rat[35].

Ushikubi et al.[36] clarified a crucial role of $EP_3$, but not $EP_1$ or $EP_4$, in fever generation by showing that $EP_3^{-/-}$ mice fail to respond to either exogenous or endogenous pyrogens. However, it remained to be further elucidated whether other EP receptors may play a role under different conditions, since the study was performed at a limited time frame (1 h after administration) and a single fixed dose of LPS (10 mg $kg^{-1}$). In the extensive studies by using different doses of LPS (1 μg $kg^{-1}$, 10 μg $kg^{-1}$, 100 μg $kg^{-1}$, or 1 mg $kg^{-1}$) and telemetrical monitoring of body temperature, Oka et al.[37] not only confirmed the central role of $PGE_2$-$EP_3$ signaling, but also clarified the role of $PGE_2$-$EP_1$ signaling in different phases of fever, at least in some limited conditions, answering the previous pharmacological studies consistently. It would be interesting to test fever responses in $EP_4$ $receptor^{-/-}$ mice because intracerebroventricular injection of an $EP_4$ receptor agonist decreases the core temperature (Tc) in rats[38]. Furthermore, it will be useful to have animals with conditional knockouts or knockins of EP receptors, which will provide opportunities to detect the responsible location where the EP receptors play a role in the complex process of fever generation.

Systemic illness also induces various adaptive responses, including tachycardia. Although inflammation-associated tachycardia has been thought to result from increased sympathetic discharge caused by inflammatory signals of the immune system, definitive proof has been lacking. Takayama et al.[39] addressed this issue. Administration of LPS to WT mice induced a biphasic increase in heart rate characterized by a transient peak (early phase) at 20 min followed by a sustained increase (late phase) that persisted for at least 100 min after LPS injection. In $TP^{-/-}$ mice, the early phase was greatly diminished, whereas the late phase was similar to that apparent in WT mice, suggesting that the $TXA_2$-TP system mediates predominantly the early phase of LPS-induced tachycardia. In $FP^{-/-}$ mice, LPS induced only the early phase of the increase in heart rate, indicating that the late phase of LPS-induced tachycardia is mediated by the $PGF_{2\alpha}$-FP system. Finally, the heart rate of LPS-treated $TP^{-/-}$ $FP^{-/-}$ mice did not differ substantially from that of vehicle-treated WT mice, indicating that $TXA_2$ and $PGF_{2\alpha}$ indeed mediate all components of LPS-induced tachycardia *in vivo*. Thus it was revealed that inflammatory tachycardia is caused by a direct action on the heart of $TXA_2$ and $PGF_{2\alpha}$ formed under systemic inflammation.

Systemic illness activates the hypothalamo-pituitary-adrenal (HPA) axis, and this response can be induced experimentally by injection of LPS. Matsuoka et al.[40] noted that although PGs had long been implicated in LPS-induced HPA axis activation, the mechanism downstream of the PGs remained unsettled. By using mice lacking each of the EP receptor subtypes ($EP_1$–$EP_4$) and an $EP_1$-selective antagonist, ONO-8713, they showed that both $EP_1$ and $EP_3$ are required for ACTH release in response to LPS. These and other findings suggested that $EP_1$- and $EP_3$-mediated neuronal pathways converge at corticotropin-releasing hormone-containing neurons in the paraventricular nucleus of the hypothalamus to induce HPA axis activation during sickness. Such regulation of HPA axis by PG receptor signaling pathways may also contribute the systemic regulation of the immune responses.

## ASTHMA

Experimental mouse models of allergic asthma established almost 10 years ago have offered new opportunities to study disease pathogenesis and to develop new therapeutics[41,42,43,44]. Although the mouse models do not reproduce all the features of human disease completely, after sensitization and respiratory tract challenges with antigen, WT mice develop a clinical syndrome that closely resembles allergic asthma, characterized by eosinophilic lung inflammation, airway hyper-responsiveness (AHR), increased IgE, mucus hypersecretion, and eventually, airway remodeling.

The roles of prostanoids in allergy have been less well defined than those in acute inflammation, in part because the effects of nonsteroidal anti-inflammatory drugs (NSAIDs) are far less marked. Nevertheless, allergic responses are associated with an increase in prostanoid formation. For example, $PGD_2$ is a major prostanoid generated by mast cells on allergen challenge and is produced abundantly in allergic diseases, such as asthma, allergic dermatitis, and conjunctivitis. Little attention had been paid to the roles of $PGD_2$ in allergy. Matsuoka et al.[45] examined this issue by subjecting $DP^{-/-}$ mice to ovalbumin-induced allergic asthma. They found a marked reduction in the airway inflammation, obstruction, and hypersensitivity in $DP^{-/-}$ animals, suggesting that $PGD_2$, acting via the DP, works as a mediator of allergy. On the other hand, Fujitani et al.[46] used transgenic (TG) mice overexpressing human lipocalin-type PGD synthase to examine the effect of overproduction of $PGD_2$ in an asthma model. They showed that these TG mice demonstrated the enhanced accumulation of eosinophils and lymphocytes in the lung, accompanied by an increase in the levels of Th2 cytokines and a chemokine. In relation with human asthma, Oguma et al.[47] demonstrated the significant association between functional genetic variants of the prostanoid DP receptor gene (PTGDR) and susceptibility to asthma. In this study, they identified several single-nucleotide polymorphisms (SNPs) in PTGDR and its vicinity. The defined SNPs are associated with the reduced gene expression with reduced transcriptional efficiency of PTGDR and a lower risk of asthma in humans. A recently developed DP receptor antagonist, S-5751, was reported to inhibited the allergic symptoms dramatically in guinea pig models of allergic rhinitis, conjunctivitis, and asthma[48]. These findings provide evidence that $PGD_2$-DP signaling mediates allergic reactions and that DP receptor antagonists may be useful in the treatment of allergic diseases.

In contrast, there are a small number of reports indicating an anti-inflammatory role of DP *in vivo*. DP activation by BW245C ameliorated the pathology of asthma[49]. Intratracheal instillation of a DP-selective agonist in combination with an immunogen inhibits airway dendritic cell (DC) migration to regional lymph nodes[50]. Although these findings seemed to be conflicting with the results obtained from the analyses of $DP^{-/-}$ mice, it is very likely that $PGD_2$-DP signaling may have an opposing effect on the asthma pathology at the different phase of disease. Indeed, they claimed the importance of $PGD_2$-DP signaling for suppressing DC migration in the initiation phase of asthma.

CRTH2 is expressed on Th2 cells and eosinophils, and mediates chemotaxis of these cells to $PGD_2$. Thus, CRTH2 is believed to be a key receptor mediating eosinophil and Th2 cell recruitment during allergic responses. To examine the role of CRTH2 *in vivo*, $CRTH2^{-/-}$ mice were generated[51]. They produce a higher amount of interleukin (IL)-5 than control littermate mice and showed enhanced eosinophilia in an asthma model, suggesting an anti-inflammatory role of $PGD_2$-CRTH2 signaling in restricting eosinophilia and allergic response *in vivo*. However, conflicting results were again observed; CRTH2 activation by 13,14-dihydro-15-keto-$PGD_2$ (DK-$PGD_2$) *in vivo* enhanced pathology of asthma in mice[49]. Since several groups have reported selective CRTH2 antagonists, it will be interesting to examine their effects on various inflammatory conditions[52,53,54,55].

The finding of proinflammatory roles of $PGD_2$-DP signaling raises a new question of why aspirin is not beneficial in allergy and can even precipitate asthmatic attacks in certain individuals. This suggests that other prostanoids normally antagonize the action of $PGD_2$, making NSAID treatment have complex effects on the disease pathway. To address this issue, Kunikata et al.[56] examined each EP receptor subtype null mice. $EP_3^{-/-}$ mice developed more pronounced allergic inflammation than that in WT or other EP subtype receptor null mice. Conversely, an $EP_3$-selective agonist suppressed the inflammation. This suppression was effective even when the agonist was administered 3 h after antigen challenge and was associated with inhibition of allergy-related gene expression. Thus, the $PGE_2$-$EP_3$ pathway is an important negative modulator of allergic reactions.

Since $EP_3$ is expressed in airway epithelial cells where the chemokines, including CCL11 and CCL17, are also expressed, $PGE_2$-$EP_3$ signaling may directly suppress the expression of these chemokine genes (Fig. 1). DP is also expressed in airway epithelial cells, suggesting that $PGD_2$-DP signaling enhances the expression of chemokine genes. Recently, it was reported that exposure to aerosolized $PGD_2$ before challenge induced the expression of macrophage-derived chemokine (MDC), a chemoattractant for Th2 cells, and accelerated Th2-type inflammation[57]. In view of molecular mechanisms of $PGD_2$-DP signaling in allergic asthma, it is reported that $PGD_2$-DP signaling is mediated via p38 MAPK, p44/42 MAPK, and PKC in a cell type-specific manner leading to NF-kB activation stimulating COX-2 gene expression[58].

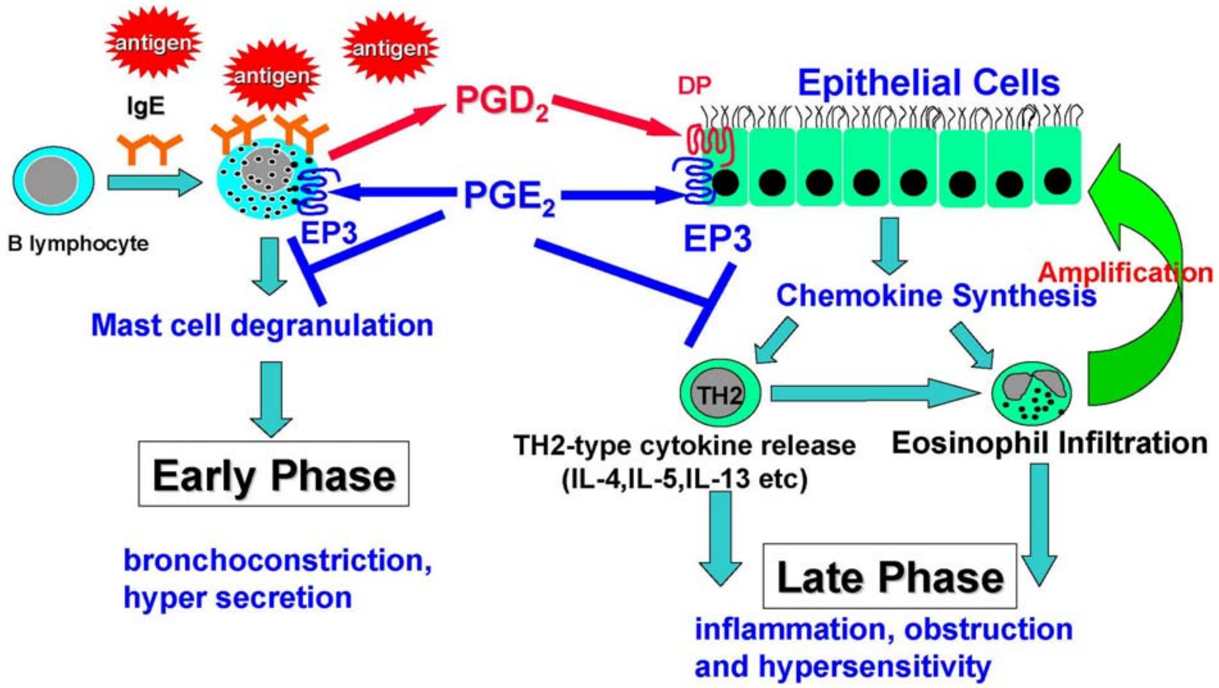

**FIGURE 1**. Schematic overview of the roles of prostanoid receptor signaling pathway in allergic asthma. Allergic inflammation consists of two phases: early-phase reaction and the late-phase reaction. In the early phase, mast cells produce $PGD_2$ on antigen challenge. $PGD_2$ acts on DP receptor in airway epithelial cells to trigger subsequent allergic reactions. $PGE_2$-$EP_3$ signaling negatively modulates allergic inflammation in both early- and the late-phase reactions (as indicated by —|). Thus, the $PGD_2$-DP pathway and the $PGE_2$-$EP_3$ pathway exert opposing actions in allergic inflammation. DP and $EP_3$ are expressed in airway epithelial cells, in which these signals may regulate synthesis and release of chemokines and cytokines.

The role of $PGI_2$-IP signaling in allergy was also examined by subjecting $IP^{-/-}$ mice to the allergic asthma model. $IP^{-/-}$ mice demonstrated increased allergic responses in the airways, which is closely related to an increase in capillary permeability[59]. They also showed a higher immune response to antigen, in terms of the production of serum IgE and IgG and T-cell reactivity, especially the Th2 response. These findings suggest a protective role for $PGI_2$-IP signaling in the allergic response.

## ATOPIC DERMATITIS

Atopic dermatitis (AD) is a chronically relapsing inflammatory disease of the skin. T cells and eosinophils are thought to play a major role in the pathogenesis of the disease[60]. Skin lesions in AD are characterized by hypertrophy of the dermis and epidermis, infiltration by T cells and eosinophils, and expression of the cytokines IL-4, IL-5, and interferon gamma (IFN-γ). Recent studies have revealed that both Th1 and Th2 cytokines may contribute to the lesions in AD[61]. A murine model of AD can be elicited by repeated epicutaneous sensitization with ovalbumin[62]. This model operates in both BALB/c and C57BL/6 mouse strains, and the skin lesions exhibit significant dermal and epidermal thickening, a cellular infiltrate consisting of T cells and eosinophils, and elevated levels of IL-4, IL-5, and IFN-γ mRNA.

Using this AD model, Angeli et al.[63] demonstrated that the potent DP agonist, BW245C, reduced the skin inflammation. It is suggested that BW245C decreases the Ag-specific T-cell activation in the skin-draining lymph nodes and disrupts the Th1/Th2 balance, probably through the increased production of the immunoregulatory cytokine IL-10, in the skin of sensitized mice. Although the anti-inflammatory effect of $PGD_2$-DP signaling in the AD model is opposite to proinflammatory effect in asthma, $PGD_2$-DP signaling activation by specific agonists may represent a strategy to control the pathological conditions of AD.

The involvement of $PGD_2$-CRTH2 signaling was also examined by murine models of skin inflammation[64]. Exogenous introduction of IgE induces immediate-type responses (ITRs) within a couple of hours, late-phase responses (LPRs) at 24 h, and very late-phase responses (vLPRs) several days after challenge. In addition, repeated challenge with haptens, the so-called chronic contact hypersensitivity (CHS) mouse model, induces an ITR followed by a late reaction with IgE elevation. The collective phenotypic appearance of these responses in mice resembles that of patients with atopic dermatitis. In these models, CRTH2$^{-/-}$ mice exhibited reduced skin responses, most markedly during vLPR. The same reduction was seen in mice administered the CRTH2 antagonist, ramatroban. Thus, the $PGD_2$-CRTH2 system plays proinflammatory roles in chronic allergic skin inflammation, and CRTH2 may represent a novel therapeutic target for treatment of human AD.

NC/Nga mice have similar pathological and behavioral features of human AD and are used as a model of the disease. Under conventional circumstances, spontaneous and persistent scratching is frequent and can lead to the onset of skin inflammation. The effects of several prostanoids and their related compounds on the scratching behavior of NC/Nga mice were examined[65]. Among them, topically applied $PGD_2$ markedly suppressed the scratching. Given the no apparent effect of DK-$PGD_2$, $PGD_2$-DP signaling is suggested to contribute to inhibiting pruritus. The DP agonist may have therapeutic effects by inhibiting pruritus of AD patients.

## ARTHRITIS

Rheumatoid arthritis (RA) is a chronic inflammatory disease of the joint characterized by inflammatory cell infiltration, synovial lining hyperplasia, and destruction of cartilage and bone. Collagen-induced arthritis (CIA) and collagen antibody-induced arthritis (CAIA) are the widely used arthritis models in the mouse[66]. CIA is induced by immunizing mice with anti-type II collagen (CII) antibody, whereas CAIA is induced by the administration of a combination of monoclonal anti-CII antibodies and LPS. CAIA can be induced in various mouse strains with rapid onset compared with that of CIA. However, the lesions of CAIA are milder and its symptoms last for a shorter duration than CIA[67]. On the other hand, although the induction of CIA is limited to a few mouse strains, such as DBA/1J, and takes about a month to develop, its lesions last for a long time and its histopathology, characterized by synovitis, pannus formation, cartilage erosion, and bone destruction in joints, is quite similar to that of human RA. Therefore, CIA is suitable for analyzing chronic joint inflammation.

The importance of PGs in the pathogenesis of RA has long been recognized through the wide use of NSAIDs for RA treatment[68,69]. Indeed, a large amount of $PGI_2$ and $PGE_2$ was detected in the synovial fluid of arthritic joints, suggesting the actions of these PGs in inflammatory sites[70]. However, how each PG works in RA has remained unknown. In the CAIA model, the importance of $EP_4$ was demonstrated, because $EP_4^{-/-}$ mice showed a profound decrease in the intensity of the inflammation, as well as a decrease in markers of joint destruction, compared to that in the other EP subtype receptor null mice[71]. In the CIA model, COX-$2^{-/-}$ mice display significant reductions in synovial inflammation and joint destruction, whereas arthritis in COX-$1^{-/-}$ mice is indistinguishable from controls[72]. Moreover, mice deficient in microsomal PGE synthase (mPGES-1) showed reduced arthritic responses[73], suggesting the importance of $PGE_2$-$EP_4$ signaling in the CIA model. However, loss or inhibition of each PGE receptor subtype alone did not affect elicitation of inflammation in CIA[74]. Simultaneous inhibition of $EP_2$ and $EP_4$ is needed to achieve a partial, but significant, suppression of CIA. Furthermore, $IP^{-/-}$ mice exhibited more significant reduction in arthritic scores compared with WT mice, despite anticollagen antibody production and complement activation similar to WT mice. Collectively, both $PGI_2$-IP and $PGE_2$-$EP_2$/$EP_4$ signaling play significant roles in the development of CIA, and this suggests that inhibition of $PGE_2$ synthesis alone may not be sufficient for suppression of RA symptoms.

There are two arms of cell populations possibly mediating the PG action in the effector mechanisms: the bone marrow–derived cell population, such as macrophages and neutrophils, and the mesenchymal cell population, such as synovial fibroblasts. It was shown that both the $PGI_2$-IP and $PGE_2$-$EP_2$/$EP_4$ signaling in synovial fibroblasts works as an amplifier of the inflammatory processes in the joint through the induction of various arthritis-related genes (Fig. 2). Whether the same signaling pathways in the bone marrow–derived cell population contribute to development of arthritis, and, if so, how they are involved in arthritis remain to be examined in bone marrow chimera in future studies.

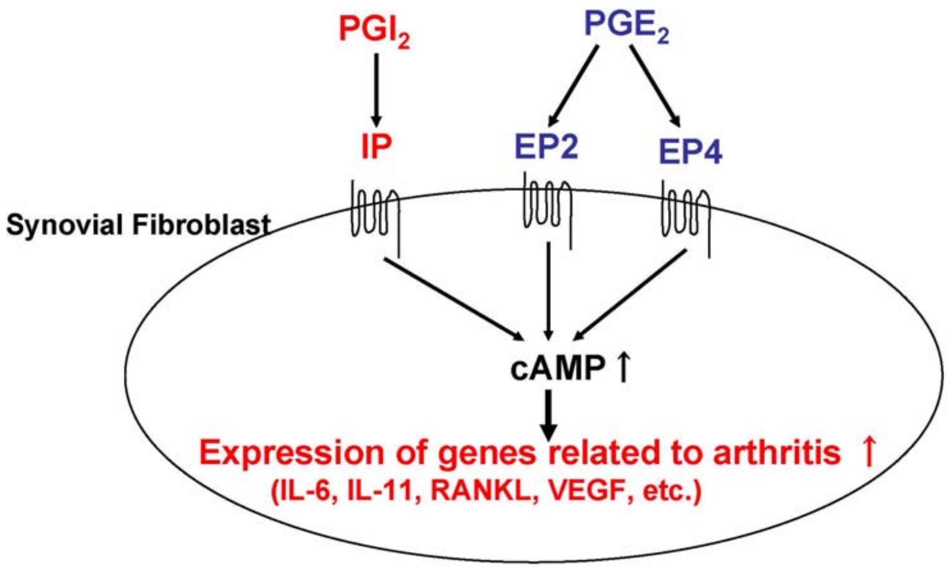

**FIGURE 2**. Schematic overview of the roles of prostanoid receptor signaling pathways in arthritis. Both the $PGI_2$-IP and $PGE_2$-$EP_2$/$EP_4$ signaling in synovial fibroblasts induces cAMP elevation, which activates these cells to stimulate the induction of various arthritis-related genes, such as IL-6, IL-11, RANKL, VEGF, etc. These mechanisms may work as an amplifier of the inflammatory responses in the arthritic joint.

## PULMONARY FIBROSIS

Pulmonary fibrosis is a common end-stage sequela of a number of acute and chronic lung diseases and characterized by inflammatory cell infiltration, fibroblast proliferation, and excess deposition of extracellular matrix proteins including collagen in the lung parenchyma. Current concepts of pathogenesis implicate dysregulated interactions between epithelial cells and mesenchymal cells. This results in the loss of normal alveolar structure and impaired lung function. As an animal model of lung fibrosis, investigators have developed the i.t. instillation of bleomycin, a chemotherapeutic agent used in the treatment of germ-line tumors and Hodgkins lymphoma, and known to induce pulmonary fibrosis with 3–5% of incidence[75,76]. In response to acute injury associated with bleomycin instillation, cellular infiltrates together with a number of blood-borne proteins enter the interstitium of the affected area of lung and results in a delayed severe fibrotic response after several days.

$PGE_2$ is the major prostanoid synthesized by lung fibroblasts[77] and neighboring alveolar epithelial cells[78,79]. Indeed, bleomycin administration increased the expression of COX-2 around 2.5-fold and resulted in a gradual increase in lung $PGE_2$ production until 14 days after injury, which slowly decreased to basal level by day 84[80]. Although earlier reports showed that continuous administration of indomethacin protected against bleomycin-induced fibrosis[81,82], $PGE_2$ has been thought to have a suppressive effect on fibrosis by inhibiting fibroblast proliferation, collagen secretion, and migration, as well as inhibiting the ability of TGF-β1 to induce fibroblast to myofibroblast differentiation[83,84,85,86]. Consistently, cultured fibroblasts from patients with idiopathic pulmonary fibrosis have an impaired ability to produce $PGE_2$, as a consequence of a reduced expression of COX-2[77,87] and mice deficient in COX-2 exhibit an enhanced response to bleomycin[88]. Recently, Moore et al.[89] have clearly demonstrated the importance of $EP_2$ in mediating suppressive effect of $PGE_2$ on fibroblast; $EP_2^{-/-}$, but not $EP_1^{-/-}$ or $EP_3^{-/-}$ mice, showed exaggerated fibrotic responses to bleomycin administration *in vivo* as compared with WT controls. They also characterized the profile of EP receptor expression on fibroblasts isolated from bleomycin-treated mice. Semiquantitative real-time RT-PCR revealed that expression levels of $EP_2$, but not $EP_1$, $EP_3$, and $EP_4$, significantly declined. Moreover, loss of $EP_2$ is sufficient to abrogate $PGE_2$ inhibition of fibroblasts in the proliferation or collagen synthesis assay, and blunted cAMP elevations in response to $PGE_2$. Thus, it is suggested that antifibrotic $PGE_2$ action can be regulated by alterations in EP receptor expression profiles during injury of the lung. However, a full understanding of the contribution to fibrogenesis by specific prostanoids and their receptors will require further efforts to explain the protective effect of indomethacin from earlier reports.

## INFLAMMATORY BOWEL DISEASE

Human inflammatory bowel disease (IBD), including Crohn's disease and ulcerative colitis, is a chronic, relapsing, and remitting condition of unknown origin, and is characterized by inflammation in the large and/or small intestine associated with diarrhea, occult blood, abdominal pain, weight loss, anemia, and leukocytosis[90]. Studies in humans have implicated impaired mucosal barrier function, pronounced innate immunity, production of proinflammatory and immunoregulatory cytokines, and the activation of CD4+ T cells in the pathogenesis of IBD. It is known that $PGE_2$ is produced abundantly in the affected intestine, and that the administration of NSAIDs often triggers and exacerbates IBD[91]. These findings indicate that $PGE_2$ works to prevent the disease initiation and progression. However, the identity of the EP receptor and mechanism of such prevention remain unknown.

As a model of IBD, experimental colitis can be induced by oral administration of dextran sodium sulfate (DSS)[92]. DSS causes acute colitis by damaging epithelial cells and, thereby, stimulating regional inflammation through production of cytokines and other inflammatory mediators. Kabashima et al.[93] subjected mice deficient in each of the eight types and subtypes of prostanoid receptors to this colitis, and examined the above issue by comparing their susceptibility to DSS treatment. Among them, only $EP_4^{-/-}$ mice developed severe colitis with 3% DSS treatment. This susceptibility in the absence of $EP_4$ signaling was confirmed by reproducing this phenotype in WT mice treated with an $EP_4$-selective antagonist. They further showed that the $EP_4$ deficiency impaired mucosal barrier function, and induced epithelial loss, crypt damage, and accumulation of neutrophils and CD4+ T cells in the colon. DNA microarray analyses in the colon of $EP_4^{-/-}$ mice demonstrated the elevated expression of genes associated with immune response and the reduced expression of genes with mucosal repair and remodeling. These findings suggest that $EP_4$ maintains intestinal homeostasis by preserving mucosal integrity and down-regulating immune response (Fig. 3). Based on these findings, a randomized placebo-controlled trial of ONO-4819CD, an $EP_4$-selective agonist, for treatment of mild to moderate ulcerative colitis is in progress. This phase II clinical trial may offer an answer and shed light on a new therapeutic approach to this disease.

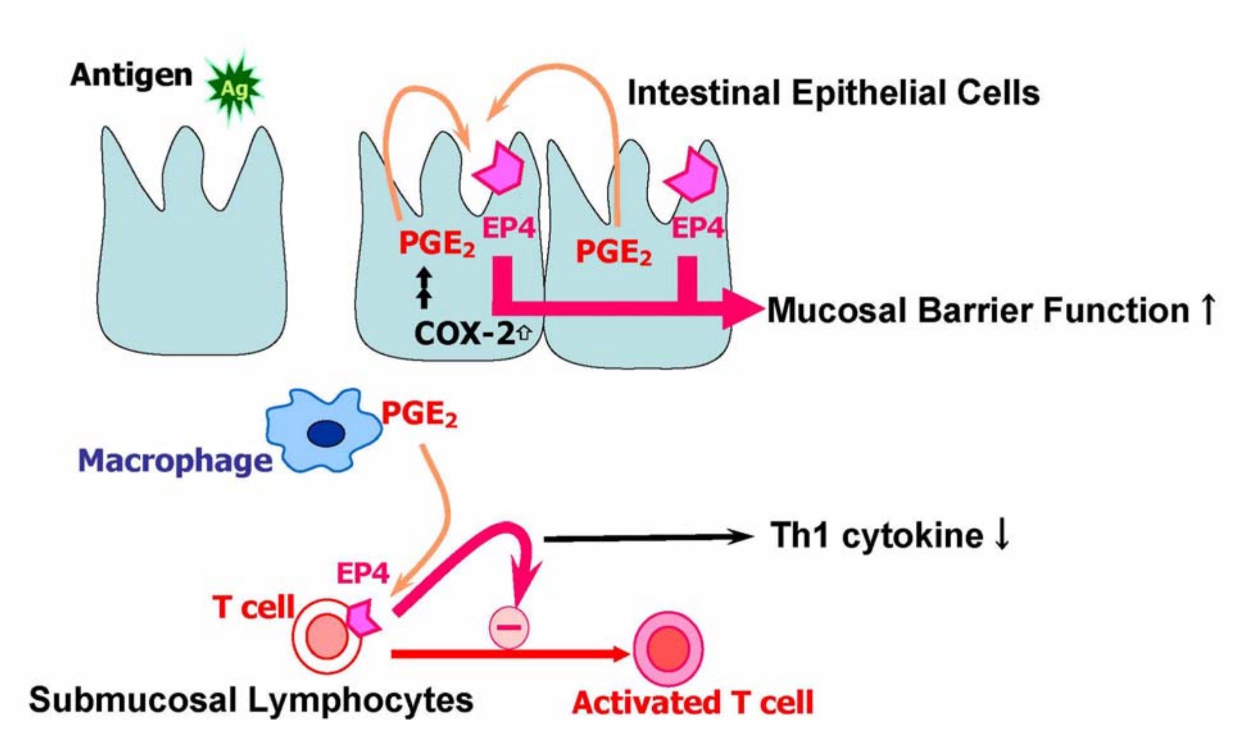

**FIGURE 3**. Schematic overview of the roles of the $PGE_2$-$EP_4$ signaling pathway in inflammatory bowel disease. The $PGE_2$-$EP_4$ signaling pathway works to keep mucosal integrity, and down-regulate the proliferation and activation of submucosal lymphocytes.

## THE EFFECTS ON DENDRITIC CELLS

Antigen exposure to the skin not only triggers immune responses, but induces acute inflammation. While $PGE_2$ is produced in substantial amounts in the skin in response to antigens and contributes to the inflammatory response, its role in induction of immune response had been unclear. Immune response in the skin is triggered by uptake of exposed antigens by Langerhans cells (LCs), a type of DC. LCs then migrate to draining lymph nodes, and activate naive T cells to initiate immune response. It is therefore likely that, if $PGE_2$ in the skin works in immune response, it acts on LCs to modulate their functions. Kabashima et al.[94] addressed this issue by analyzing mice deficient in each EP receptor subtype, and found that, while LCs express all four EP subtypes, their migration to regional lymph nodes after antigen uptake was decreased only in $EP_4^{-/-}$ mice. Impairment in migration was reproduced in WT mice treated with an $EP_4$ antagonist, whereas an $EP_4$ agonist promoted the migration of LCs. $EP_4$ stimulation further increased expression of costimulatory molecules in LCs, and enhanced their ability to stimulate T cells in the mixed lymphocyte reaction (MLR) *in vitro*. These results indicate that $PGE_2$-$EP_4$ signaling promotes the migration and maturation of LCs and facilitates initiation of skin immune responses. Consistent with this hypothesis, CHS to antigen was impaired in $EP_4^{-/-}$ mice as well as in WT mice treated with the $EP_4$ antagonist during sensitization.

While the above work elucidated the role of the $PGE_2$-$EP_4$ signaling in initiation of immune response, this finding appears to contrast with the observation that NSAIDs that block PG biosynthesis generally do not exhibit modulatory effects on such responses. This apparent discrepancy might be explained if we hypothesize that other prostanoid signaling than $PGE_2$-$EP_4$ signaling exert opposing actions in immune responses. Indeed, $PGD_2$ was recently shown to be produced in the skin and inhibit LCs migration during *Schistosoma mansoni* infection[63]. As pathogens including protozoan parasites and fungi have been shown to synthesize $PGD_2$, it was hypothesized that $PGD_2$ may modulate the host inflammatory and immune responses[95,96]. It was demonstrated that the production of $PGD_2$ by the skin stage of *S. mansoni* inhibits the migration of epidermal LCs and the subsequent accumulation of DCs in the draining lymph nodes. Since $DP^{-/-}$ mice restored the LCs migration and developed a less severe inflammation, it was proposed that the Schistosome use $PGD_2$-DP signaling to evade host immune defenses by disrupting the appropriate Th1/Th2 balance of the host immune responses during infection.

## THE EFFECTS ON THYMOCYTE DEVELOPMENT

The effects of prostanoid on T-cell development have been reported. In the thymus, $TXA_2$-TP and $PGE_2$-EPs signaling are implicated to modulate T-cell development and maturation. TP receptors are expressed at high levels in the thymus, most prominently in immature thymocyte populations, and stimulation of TP receptors on these cells promotes apoptosis[97]. These data suggest that TP receptors on thymocytes might play a role in negative selection of maturing T lymphocytes. These actions may be relevant to *in vivo* immune responses, since Remuzzi and associates have found that the actions of TP receptors in the thymus may be critical for the development of tolerance to renal allografts[98].

On the other hand, there is a report that $PGE_2$ protects immature CD4+CD8+ (double positive) thymocytes from apoptosis[99]. This action appears to be mediated by cAMP, suggesting involvement of $EP_2$ or $EP_4$ receptors. Rocca et al.[100] used fetal thymic organ culture and examined effects on thymocyte differentiation of selective COX-1 or COX-2 inhibitors, as well as disruption of a gene for either isoform. They found that COX-1 inhibitors, added to the culture, significantly suppressed transition of CD4−CD8− double-negative thymocytes to CD4+CD8+ double-positive cells, while COX-2-selective inhibitors impaired the differentiation of CD4+CD8+ cells to CD4+ single-positive T cells. The effect of the COX-2 inhibitors was reproduced in the thymus culture from COX-$2^{-/-}$ mice, and the suppression of the double-negative to double-positive cells was also noted, albeit weakly, in the thymi of COX-$1^{-/-}$ mice. They also reported that PGE analogs with the $EP_2$ agonist activity could rescue the inhibition on CD4+CD8+ double-positive cell maturation by COX-1 inhibitors, and those with the $EP_1$ agonist activity reversed the effects of COX-2 inhibitors. These results suggest that both COX isozymes and the $PGE_2$-$EP_1$/$EP_2$ signaling are required for the efficient maturation of the T-cell lineage in fetal thymus.

## THE EFFECTS ON THE DEVELOPMENT AND FUNCTION OF MATURE LYMPHOCYTES

A large number of reports have described involvement of prostanoids in the development and function of mature lymphocytes. $PGE_2$-EPs signaling are suggested to inhibit a wide range of T- and B-cell functions[101]. Conversely, $TXA_2$ may promote T-cell activation and proliferation, and facilitate the development of effecter T cells[102]. Using the mixed lymphocyte reaction (MLR) as a model cellular immune response on mice lacking each EP receptor subtype, it was demonstrated that $PGE_2$-$EP_2$ receptor signaling directly inhibits T-cell proliferation, while $PGE_2$-$EP_2$/$EP_4$ receptor signaling regulates antigen presenting cell functions[103]. Thus, $PGE_2$ regulates cellular immune responses through distinct EP receptors on different immune cell populations. This finding is consistent with previous studies suggesting that the inhibitory effects of $PGE_2$ were related to accumulation of intracellular cAMP, implicating a G-protein coupled receptor such as $EP_2$ or $EP_4$[104]. They also claimed proinflammatory actions of $TXA_2$-TP signaling in cellular immune responses from their other study on $TP^{-/-}$ mice[105].

On the contrary, negative regulation of T-cell functions by $TXA_2$-TP signaling was demonstrated[106]. Physical interaction of T cells and DCs is essential for T-cell proliferation and differentiation. Kabashima et al.[106] have shown that DCs produce $TXA_2$, whereas naive T cells express TP. In this study, I-BOP, a TP agonist, enhances random cell movement of naïve, but not memory, T cells, impairs DC-T cell adhesion, and inhibits DC-dependent proliferation of T cells. Immune responses to foreign antigens are enhanced in TP-deficient mice, which also develop marked lymphadenopathy with age. Similar immune responses were seen in WT mice treated with a TP antagonist during the sensitization period. These results clearly demonstrated a novel role of the $TXA_2$-TP signaling in modulation of immunity to foreign antigens by negatively regulating DC-T cell interactions.

Naturally occurring CD4+CD25+ regulatory T cells (Treg) play a pivotal role in suppressing immune responses and maintaining tolerance. The identification of molecules controlling Treg differentiation and function is important in understanding host immune responses in malignancy and autoimmunity. It was suggested that $PGE_2$ enhances the Treg function by inducing the T reg cell-specific transcription factor forkhead/winged helix transcription factor gene (FOXP3) in CD4+CD25- T cells and significantly up-regulated its expression in CD4+CD25+ Treg cells in human[107]. It was also reported that human adaptive Treg cells, expressing COX-2, produce $PGE_2$ and suppress effecter T-cell responses[108]. Thus, autocrine and paracrine effects of $PGE_2$ produced by Treg cells may be responsible for the mechanism used by these cells to suppress effecter T cells. It remains for the future studies to determine responsible prostanoid receptor signaling pathways for these inhibitory effects.

## THE EFFECT ON B LYMPHOCYTES CLASS SWITCHING

$PGE_2$ has been reported to inhibit numerous immunologic events of B and T lymphocytes, including IL-2 production and receptor expression in T cells, cell activation events, and immunoglobulin class switching in B cells. $PGE_2$ can inhibit the production of the Th1 cytokines IL-2 and IFN-γ, shifting the balance in favor of a Th2 response that enhances IL-4 and IL-5 production and facilitates immunoglobulin class switching to IgE[109]. However, other studies have suggested that $PGE_2$ inhibits Th2 cytokine secretion and IgE production by B cells[110]. Furthermore, it was also demonstrated that $PGE_2$ promotes total IgG1 secretion and the number of IgG1-secreting cells[111]. Although the regulation of IgE and IgG1 are known to differ, agents that elevate cAMP mimic the effect of $PGE_2$ on B-cell responses, suggesting the involvement of $PGE_2$-$EP_2$/$EP_4$ signaling in immunoglobulin class switching[112]. Future studies using mice deficient in each EP receptor subtype and selective agonists and antagonist should clarify the role of $PGE_2$ in immunoglobulin class switching *in vivo*.

## CONCLUSION

In this review, we have summarized the recent findings of the roles of prostanoid receptor signaling in immune and inflammatory responses. These finding were mainly derived from the examinations of the gene knockout mice in various kinds of animal models of human disease. Although species differences between human and mouse should be considered cautiously and extrapolation from the mouse models to human pathologies must be performed with some reservation, they provide a considerable amount of information and suggest that these signaling pathways play important roles to modulate their physiological and pathophysiological conditions. They also provide useful and significant data for development of new therapeutics that selectively stimulate or inhibit each prostanoid action. Indeed, some of the knockout mouse phenotypes have been confirmed and reproduced by the use of newly developed compounds selective to each type of receptors. However, they also revealed that these receptor signaling pathways frequently have opposing actions through complex interaction mechanisms between different prostanoids, target cells, phase of reaction, and so on. Indeed, they can produce both pro- and anti-inflammatory actions in the context-dependent manner. The importance of each of these signaling pathways can be altered depending upon the inflammatory stimulus, the type of prostanoids predominantly produced, and the profile of prostanoid receptor expressions. More specific studies will be required to address these issues. Together with recently developed specific pharmacological reagents, tissue-specific inducible gene knockout animals will be extremely powerful tools to dissect the precise mechanisms for elucidating the roles of these signaling pathways in each disease condition. These studies certainly facilitate a search for new applications of the highly selective agonists and antagonists to human disease conditions.

## ACKNOWLEDGMENTS

This work was supported in part by Grants-in-Aid for Scientific Research from the Ministry of Education Culture Sports Science and Technology of Japan; by a grant from the National Institute of Biomedical Innovation of Japan; and by grants from the Kowa Life Science Foundation, the Takeda Scientific Foundation, and the ONO Research Foundation.
